# Association of gait speed and handgrip strength with falls in older adults: the role of cognition

**DOI:** 10.55730/1300-0144.5882

**Published:** 2024-06-17

**Authors:** Neslihan KAYAHAN SATIŞ, Sultan KESKİN DEMİRCAN, Mehmet İlkin NAHARCI

**Affiliations:** Division of Geriatrics, Department of Internal Medicine, Gülhane Research and Training Hospital, University of Health Sciences, Ankara, Turkiye

**Keywords:** Fall, cognitive impairment, gait speed, handgrip strength

## Abstract

**Background/aim:**

Fall risk assessment is crucial for older adults because falls are associated with morbidity and mortality. This study investigated the relationship of gait speed (GS) and handgrip strength (HGS) with falls and assessed whether cognition mediates this causality.

**Materials and methods:**

The study was conducted in a tertiary referral geriatric outpatient clinic. The physical performance of participants was evaluated by GS and HGS. All falls in the previous year were noted and factors associated with falls were analyzed using multivariate regression analysis.

**Results:**

A total of 1018 older adults with a mean age of 78.8 ± 7.2 years, 64.2% of whom were female, were stratified into two groups: those who were cognitively impaired (n = 331) and those who were cognitively healthy (n = 660). In the study population, 22.8% (n = 226) had a history of falls in the previous year. The rates of low GS and HGS were 29.1% and 80.6%, respectively. After adjusting for confounding factors, low GS (OR = 2.01, 95% CI: 1.10–3.77, p = 0.019), low HGS (OR = 3.57, 95% CI: 1.10–11.35, p = 0.038), and low GS plus low HGS (OR = 4.52, 95% CI: 1.14–15.78, p = 0.024) in the cognitively impaired group and low GS (OR = 2.13, 95% CI: 1.39–3.52, p = 0.003) in the cognitively healthy group were independently associated with falls.

**Conclusion:**

GS is an efficient and practical assessment tool for identifying older adults at risk of falls regardless of their cognitive status.

## 1. Introduction

Falls are a common health problem, causing reduced quality of life and functionality, anxiety about falling, and increased morbidity and mortality in older adults. Approximately one in three people aged >65 years among community dwellers and one in two people aged >80 years fall one or more times within a year, and half of these falls are repeated falls [1–3]. An increase in the likelihood of falls in older adults occurs with the onset of initial mobility impairment. One of the risk factors responsible for the progression of mobility difficulties at an advanced age is decreased cognition [4–6].

Poor cognitive function increases the risk of falls and injuries caused by falls, especially in older individuals with dementia, who are eight times more likely to have these comorbidities than their cognitively healthy peers [7]. Cognitive impairment may lead to disturbances, especially in executive function, attention, inhibitory control, and working memory, which are all necessary to avoid falls [8–10]. Thus, maintaining healthy cognition in vulnerable older adults is essential to reduce the progression of functional decline and prevent disability [3,11–13].

Handgrip strength (HGS) and gait speed (GS), which are physical parameters that change with age, are closely related to each other, reflecting muscle function and future fall risk [14–18]. While GS is usually a measure of functionality or mobility, HGS is a measure of muscle strength. Recent analyses have shown that weak HGS and slow GS are associated with a decrease in cognitive function in different domains, and muscle strength training is recommended to improve cognitive function [10,19,20]. Although falls are known to correlate with a decrease in muscle function [21–25], information on the role of cognition in these associations in individuals without dementia is limited. Our aim is to examine the relationship between falls and frailty according to cognitive status. This study seeks to better understand how cognitive function influences the association between falls and muscle function.

## 2. Materials and methods

### 2.1. Participants

This study was a longitudinal cohort study of consecutive older adults (≥65 years) recruited from a geriatric outpatient clinic at a tertiary referral center. From March 2020 to March 2023, a total of 10,021 admissions were evaluated for study enrollment. Individuals with psychotic disorders, previous diagnoses of any type of dementia, orthopedic or rheumatic conditions that impair mobility, homebound status, neurological disorders other than Parkinson disease, severe systemic or infective disease, visual or sensorial disability, or a lack of a cognitive assessment were excluded at first admission (Figure). All participants were capable of walking autonomously, with or without assistive equipment. All participants provided written informed consent and the local ethics board approved the study (2020-7/1).

### 2.2. Assessment of cognition

Routine mental assessment by a panel of two internists and experienced geriatricians included a review of the medical history and medications based on information from the patient and an informant, a physical examination, laboratory tests, and neuroimaging if needed. The screening test used for cognitive status was the Mini-Mental State Examination (MMSE), a 30-point tool (higher scores indicate better performance) that assesses attention, memory, language, orientation, and visual-spatial skills [26]. Following a comprehensive evaluation, clinicians determined whether the individuals had delirium, any type of dementia, or neuropsychiatric disorders, and patients with these conditions were also excluded from the study [27–29]. The final number of participants in this study was 991. Subsequently, participants without dementia were divided into groups according to their MMSE scores: those with scores of ≥27 were categorized as cognitively healthy and those with scores of ≤26 as cognitively impaired (Figure) [30]. The validated Turkish version of the MMSE was used, which was prepared for administration among individuals with less than 5 years of education [31].

### 2.3. Measurements

The usual GS was measured indoors on a flat surface. Participants were asked to walk in a normal rhythm from the start of standing. The walkway was 7.51 m long, including an acceleration zone of 1.52 m, a central test zone of 4.57 m, and a deceleration zone of 1.52 m. A chronometer was used to measure the time. The test was performed twice with 1 min of rest between each attempt. The two attempts were averaged and documented. The distance covered (4.57 m) was divided by the walking time to calculate walking speed (m/s). According to the Fried criteria, the participants were classified as having normal or low GS, which was further stratified based on sex and height [32]. HGS was measured using a manual dynamometer (Jamar, JLW Instruments, Chicago/USA). It had resolution of 2 kilogram/force (kgf) and a scale of 0–90 kgf. The participant was standing, the forearm was unsupported, and the spine was straight. Shoulders were in the adduction position and neutrally rotated. The forearm was situated in the midprone position, with the elbow flexed to 90° and the wrist in neutral with 30° of extension. The participants performed the procedure with maximum force for approximately 5 s with their dominant hand. A period of 1 min between measurements was also recorded. The average of three assessments was taken into consideration [33]. We classified HGS, depending on sex and body mass index (BMI), as normal or low according to the criteria of Fried et al. [32].

### 2.4. Outcome variable

The outcome of interest was the fall history of participants. Falls were noted as any occurrence of a person inadvertently resting on or below the ground [34]. Participants and/or their relatives, if any, were asked whether there had been a fall and the number of falls in the previous year. All falls during the previous 12 months were recorded.

### 2.5. Demographics and comorbidities

Sociodemographic characteristics of the patients (sex, age, BMI, marital status, education) and comorbid conditions such as coronary artery disease, diabetes mellitus, hypertension, chronic obstructive lung disease, cerebrovascular disease, Parkinson disease, depression, osteoporosis, and urinary incontinence were noted. Comorbidities were determined through patient self-reports, interviews with informants, and electronic records. All of these factors may contribute to functional impairment and cognitive changes among patients [35–38]. A lower level of education was described as ≤5 years. The number of drugs was noted and the concomitant usage of five or more drugs was defined as polypharmacy [39]. The anticholinergic burden was determined using the Anticholinergic Cognitive Burden (ACB) scale, and high exposure was classified as a score of ≥1 [40]. The Barthel index was used for functional assessment and functional impairment was defined by <90 points (range: 0–100) [41]. The Mini-Nutritional Assessment-Short Form (MNA-SF) (range: 0–14) was used to determine nutritional status [42].

### 2.6. Statisticsv

IBM SPSS Statistics (IBM Corp., Armonk, NY, USA) was used for statistical analysis. Variables were presented as absolute number and percentage, mean ± standard deviation, and median (minimum–maximum), as appropriate. The Student t-test or Mann–Whitney U test was used for comparisons of continuous variables. The Kolmogorov–Smirnov test was used to determine data distribution. Categorical variables were compared using the chi-square test. Multivariate analyses were conducted with falls as the dependent variable and GS, HGS, and their combinations (normal GS and normal HGS, either low GS or low HGS, and low GS plus low HGS) as the predictive variables. In univariate analysis, variables with statistical significance (p ≤ 0.05) and clinically relevant variables (sex) were selected to construct a multivariate regression model. Subsequently, three sequential models were created for multiple testing of the cognitively impaired and healthy groups. These included Model 1 (unadjusted), Model 2 (adjusted for age and sex), and Model 3 for the cognitively impaired group (factors in Model 2 plus cerebrovascular disease, depression, urinary incontinence, polypharmacy, anticholinergic burden, and functional impairment) and the cognitively healthy group (factors in Model 2 plus marital status, duration of education, chronic obstructive lung disease, Parkinson disease, depression, osteoporosis, urinary incontinence, and anticholinergic burden). The Hosmer–Lemeshow test was used to evaluate the fitness of the model. Values of p < 0.05 were accepted as statistically significant.

## 3. Results

Among the eligible participants (n = 991), 22.8% (n = 226) had a history of falls in the previous year. The mean age was 78.8 ± 7.2 years and women constituted the majority (64.2%). The rates of low GS and HGS were 29.1% and 80.6%, respectively. The mean MMSE score was 26.8 ± 3.0, and participants with cognitive impairment constituted 33.4% of the population (n = 331). The demographics and disease characteristics of all participants according to cognitive status and fall history are shown in Table 1.

In the cognitively impaired group, fallers were older (p < 0.001) and had more cerebrovascular disease (p = 0.003), depression (p = 0.019), urinary incontinence (p < 0.001), number of drugs (p < 0.001), polypharmacy (p < 0.001), ACB score of ≥1 (p < 0.001), and functional impairment (p = 0.029) than nonfallers, as well as lower GS (p < 0.001) and HGS (p < 0.001). Other variables did not differ in this group according to the history of falls (Table 1). In the cognitively healthy group, fallers were older (p < 0.001), less likely to be married (p = 0.013), and had higher rates of ≤5 years of education (p = 0.014), chronic obstructive lung disease (p = 0.024), depression (p < 0.001), osteoporosis (p < 0.001), urinary incontinence (p < 0.001), number of drugs (p < 0.001), and ACB score of ≥1 (p = 0.003), as well as lower MMSE scores (p = 0.025), MNA scores (p < 0.001), GS (p < 0.001), and HGS (p < 0.001) compared to nonfallers. Other variables did not differ in this group according to the history of falls (Table 1). As a complement to our analyses, the Supplementary Table includes the demographics and disease characteristics of all participants, stratified by fall status.

### 3.1. Association of GS and HGS with falls based on cognition

In the cognitively impaired group, low GS, low HGS, and low GS plus low HGS in the unadjusted analysis (Model 1) were associated with falls. After adjusting for age and sex (Model 2), these relationships remained significant. In the full model after adjusting for all confounders (Model 3), low GS (OR = 2.01, 95% CI: 1.10–3.77, p = 0.019), low HGS (OR = 3.57, 95% CI: 1.10–11.35, p = 0.038), and low GS plus low HGS (OR = 4.52, 95% CI: 1.14–15.78, p = 0.024) were still associated with falls (Table 2). In the cognitively healthy group, low GS and low GS plus low HGS in the unadjusted model (Model 1) were associated with falls, whereas low HGS alone was not. These relationships were similar after adjusting for age and sex (Model 2). In the full model after adjusting for all confounders (Model 3), only low GS (OR = 2.13, 95% CI: 1.39–3.52, p = 0.003) was associated with falls (Table 3). The Hosmer–Lemeshow test for Model 3 in the cognitively impaired and healthy groups provided chi-square values of 13.789 and 8.956, respectively, which were insignificant (p = 0.102 and p = 0.378), revealing that the models were a good fit for the data.

## 4. Discussion

Understanding the predictive factors of falls, which are significant causes of mortality and morbidity in older adults, has a potential impact on patient prognosis. Our study focused on the possible effects of isolated and concomitant evaluation of functional measurements on falls in terms of the cognitive status of people aged 65 and older, which was investigated for the first time in the literature to the best of our knowledge. The main findings showed that low GS, low HGS, and low GS plus low HGS in the cognitively impaired group and only low GS in the cognitively healthy group were significantly associated with falls, even after controlling for confounding variables. Moreover, low GS plus low HGS in individuals with cognitive impairment was more strongly related to falls.

Our data show the association between falls and both GS and HGS, especially in individuals with poor cognition, in line with the findings of previous studies. Previous studies revealed that cognitive performance influences balance, mobility, and fall risk in older adults [8,9,12,13,43–47], and significant interaction with physical function could dramatically accelerate falls at advanced ages [48]. Despite an abundance of studies showing that falls increase in older adults with cognitive impairment [12,13,43–47], few studies have examined the effect of the coexistence of cognitive impairment and physical weakness on falls. Consistent with previous findings, we found that poor GS and HGS predisposed older adults with cognitive impairment to falls [48,49]. Of note, we determined that participants with low HGS plus low GS in the cognitively impaired group were at greater risk of falling than those with declines in single measures of these parameters, suggesting that observing both physical components allows the best assessment of fall risk in this vulnerable population.

Our findings also suggest an association between low GS and falls in cognitively healthy individuals, which is consistent with studies related to gait performance and balance disorders [50–52]. Conversely, the results of a small-sample study (n = 135) involving only older adult women showed that low GS was not significantly associated with falls [53]. On the other hand, the MOBILIZE Boston Study, a population-based longitudinal cohort, found a nonlinear U-shaped correlation between GS and subsequent falls such that those with faster or slower GS than the specified value were at the highest risk of falls [54]. However, the cutoff points used in that study were different from those used in our study. Additionally, similar to other studies, we found that HGS was not predictive of falls in the cognitively healthy group [50,55]. A metaanalysis of prospective studies showed that upper-extremity weakness, as a predictor of lower-extremity weakness, may be a reason for falls in both institutionalized and community-dwelling subjects, partially supporting our findings [25]. Furthermore, our findings indicate that adding low GS to low HGS does not significantly contribute to the prediction of falls in the healthy group. Clinically, individuals with intact cognitive function walking slowly may be ideal nominees for multifactorial interventions aimed at reducing falls and related injuries. In addition, the analysis of cognitive assessments in older adults with a history of falls revealed different previously unaddressed associations with functional measures.

GS is a measure of muscle strength as well as motor control and balance. Chronic comorbid conditions and psychotropic drugs can affect any component of GS, causing falls even in cognitively healthy individuals [56]. In contrast, HGS is a more specific measure of physical capability and remains intact before the development of cognitive impairment [56]. Pathophysiological processes such as decreased sex hormones, elevated chronic inflammation markers, oxidative stress, malnutrition, and physical inactivity, which cause cognitive decline, also play roles in the decrease in HGS [12,57,58]. As the HGS level decreases, its relationship with falls increases, which may clarify the relationship between HGS and falls in the cognitively impaired group in our study [59]. Furthermore, GS is related to reduced gray and white matter volumes in several parts of the brain, whereas HGS is associated with the dorsal and ventral parts of the premotor cortex and the primary and supplementary motor areas, which are thought to control executive function, attention, decision-making, comprehension, and working memory [60]. Our findings further support the role of these neuroanatomical differences in the connection between HGS and falls only in subjects with cognitive impairment.

GS and HGS are crucial parameters in defining frailty [21]. The relationship between frailty and falls is well documented [21,32], but individuals with cognitive impairment have been shown to be more vulnerable to falls [61]. HGS and GS are also utilized in the assessment of sarcopenia, and sarcopenia is closely connected to a greater risk of falling [15]. Studies have shown that severe sarcopenia when present together with a physical performance impairment, such as a decreased walking speed, causes an increase in falls [62,63]. Furthermore, in a recent metaanalysis, sarcopenia was found to occur 1.88 times more frequently in those with cognitive impairment and a history of falls [64]. Although the primary endpoints of these studies were different from ours, they provided data that validated the results of our study. In summary, these findings demonstrate the connection between physical and cognitive status in relation to falls.

Our study had several limitations and strengths. First, the cross-sectional study design did not identify causality. Due to being conducted in a single center, the study’s generalizability is limited. Second, many clinical parameters were exhaustively included in the study, but unmeasured variables in the analysis may have been neglected as potential confounders. We used patient history for the evaluation of falls; however, falls occurring during follow-up could be a better indicator for determining risk factors. Another limitation is the fact that the use of patient/caregiver memory to estimate falls in the previous year is not fully reliable because of recall bias. The most important strength of our study is that functional and mental evaluations were conducted through a standardized comprehensive geriatric assessment, which improved the quality of the data collected and the overall quality of the study. Instead of using a standard cutoff point, patient evaluation was based on classifying and correcting GS and HGS according to sex, BMI, and height, which is another positive aspect. Our study was conducted using a fairly large cohort. Furthermore, the functional measurements we chose can be quickly and easily utilized in outpatient clinics to establish fall prevention strategies based on cognitive status and decrease the probability of falls.

Our study provides evidence of the association between functional parameters moderated by cognitive function and falls. Our findings indicate a cross-sectional association between GS and falls regardless of cognitive function in older adults, and a combined assessment of both HGS and GS may improve the prediction of subsequent falls in older adults with cognitive impairment. Further large-sample longitudinal studies are required to clarify the predictive impact of gait and muscle strength among cognitively different populations at a high risk of falls.

## Supplementary ınformation

**Supplement 1:** Characteristics of the participants in terms of fall history. BMI: Body mass index, MMSE: Mini mental state examination, MNA-SF: Mini nutritional assessment short form. Results that are statistically significant are highlighted in bold. There are individuals with missing data.

	Overall N:991	Fallers (n=226)	Nonfallers (n=765)	p
Age (years), mean (SD)	78.8 (7.2)	81.1 (6.7)	78.1 (7.2)	**<0.001**
Sex (female), n (%)	637 (64.2)	162 (71.6)	475 (62.1)	**0.034**
BMI, mean (SD)*	28.5 (4.9)	29.0 (5.4)	28.3 (4.7)	
Marital status (married), n (%)	592 (59.7)	109 (48.2)	483 (63.1)	**<0.001**
Education time (≤5 years), n (%)	623 (67.1)	458 (59.9)	458 (73.0)	**<0.001**
Current smokers, n (%)	61 (6.2))	17 (7.5)	44 (5.8)	0.331
Current alcohol users, n (%)	14 (1.4)	2 (0.9)	12 (1.6)	0.444
Comorbidities, n (%)				
Hypertension, n (%)	735 (74.2)	179 (79.2)	556 (72.7)	0.060
Diabetes mellitus, n (%)	346 (34.9)	83 (36.7)	263 (34.4)	0.568
Coronary artery disease, n (%)	263 (26.5)	60 (26.5)	203 (26.5)	1.000
Chronic obstructive lung disease, n (%)	102 (10.3)	32 (14.2)	70 (9.2)	**0.040**
Cerebrovascular disease, n (%)	53 (5.3)	21 (9.3)	32 (4.2)	**0.005**
Depression, n (%)	324 (32.7)	103 (45.6)	221 (28.9)	**<0.001**
Osteoporosis, n (%)	201 (20.3)	66 (29.2)	135 (17.6)	**<0.001**
Urinary incontinence, n (%)	290 (29.3)	113 (50)	177 (23.1)	**<0.001**
Polypharmacy (≥5 drugs), n (%)	440 (44.4)	125 (55.3)	315 (41.2)	**<0.001**
Anticholinergic burden (≥1), n (%)	326 (32.9)	106 (46.9)	220 (28.8)	**<0.001**
MMSE score, mean (SD)	26.8 (3.0)	26.3 (2.8)	27.0 (3.0)	**0.002**
Cognitive Impairment	331 (33.4)	99 (43.8)	232 (30.3)	**<0.001**
Functional impairment, n (%)	94 (9.5)	33 (14.7)	61 (8.0)	**0.004**
MNA-SF, mean*	12.5 (1.9)	12.0 (2.2)	12.6 (1.8)	**<0.001**
Gait speed, mean (SD)	0.83 (0.3)	26.3 (2.9)	27.0 (3.0)	**<0.001**
Low gait speed, n (%)*	281 (29.1)	109 (48.4)	172 (23.0)	**<0.001**
Handgrip strength, mean (SD)(range)*	17.3 (8.0)	14.4 (7.1)	18.2 (8.1)	**<0.001**
Low handgrip strength, n (%)*	779 (80.6)	196 (88.7)	583 (78.2)	**0.001**

## Figures and Tables

**Figure:**
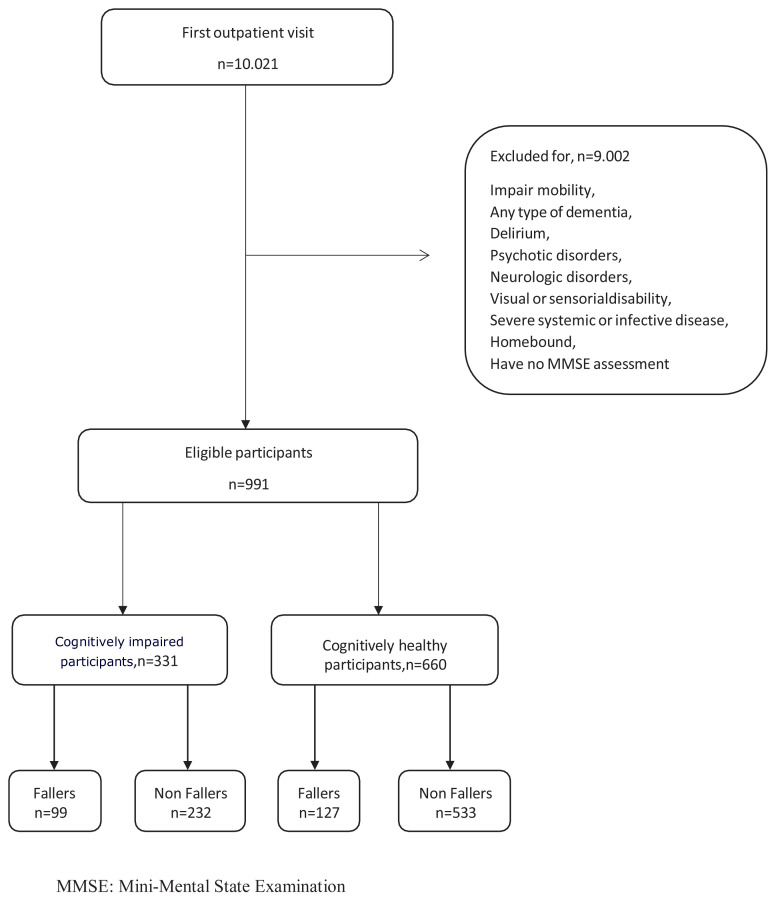
Flow chart of patient selection process.

**Table 1:** Characteristics of the participants in terms of cognitive status and fall history. BMI: body mass index, MMSE: mini mental state examination, MNA-SF: mini nutritional assessment short form. Results that are statistically significant are highlighted in bold. There are individuals with missing data.

Variables	Overall (n=991)	Cognitively impaired (n=331)			Cognitively healthy (n=660)		
		Fallers (n=99)	Nonfallers (n=232)	p	Fallers (n=127)	Nonfallers (n=533)	p
Age (years), mean (SD)	78.8 (7.2)	83.1 (6.2)	80.3 (6.9)	**<0.001**	79.5 (6.7)	77.1 (7.1)	**<0.001**
Sex (female), n (%)	637 (64.2)	77 (77.7)	165 (71.1)	0.242	85 (66.9)	310 (58.1)	0.121
BMI, mean (SD)*	28.5 (4.9)	29.0 (5.4)	27.9 (4.8)	0.093	29.0 (5.3)	28.5 (4.7)	0.357
Marital status (married), n (%)	592 (59.7)	37 (37.4)	119 (51.3)	0.037	72 (56.7)	364 (68.3)	**0.013**
Education time (≤5 years), n (%)	623 (67.1)	85 (85.9)	187 (80.6)	0.253	47 (37.0)	262 (49.2)	**0.014**
Current smokers, n (%)	61 (6.2)	6 (6.1)	11 (4.7)	0.619	11 (8.7)	33 (6.2)	0.316
Current alcohol users, n (%)	14 (1.4)	-	5 (2.2)	0.141	2 (1.6)	7 (1.3)	0.819
Comorbidities, n (%)							
Hypertension, n (%)	735 (74.2)	80 (80.8)	171 (737)	0.167	99 (78.0)	385 (72.2)	0.190
Diabetes mellitus, n (%)	346 (34.9)	44 (44.4)	75 (32.3)	0.061	39 (30.7)	188 (35.3)	0.331
Coronary artery disease, n (%)	263 (26.5)	31 (31.3)	72 (31)	0.960	29 (22.8)	131 (24.6)	0.680
Chronic obstructive lung disease, n (%)	102 (10.3)	11 (11.1)	19 (8.2)	0.397	21 (16.5)	51 (9.6)	**0.024**
Cerebrovascular disease, n (%)	53 (5.3)	14 (14.1)	11 (4.7)	**0.003**	7 (5.5)	21 (3.9)	0.430
Depression, n (%)	324 (32.7)	44 (44.4)	72 (31)	**0.019**	59 (46.5)	149 (28.0)	**<0.001**
Osteoporosis, n (%)	201 (20.3)	27 (27.3)	47 (20.3)	0.161	39 (30.7)	88 (16.5)	**<0.001**
Urinary incontinence, n (%)	290 (29.3)	56 (56.6)	56 (24.1)	**<0.001**	57 (44.9)	121 (22.7)	**<0.001**
Polypharmacy (≥5 drugs), n (%)	440 (44.4)	60 (60.6)	88 (37.9)	**<0.001**	65 (51.2)	227 (42.6)	0.080
Anticholinergic burden (≥1), n (%)	326 (32.9)	54 (54.5)	74 (31.9)	**<0.001**	52 (40.9)	146 (27.4)	**0.003**
MMSE score, mean (SD)	26.8 (3.0)	23.4 (3.0)	23.6 (2.2)	0.465	28.3 (1.0)	28.6 (1.0)	**0.025**
Functional impairment, n (%)	94 (9.5)	29 (29.6)	43 (18.7)	**0.029**	4 (3.1)	18 (3.4)	0.890
MNA-SF, mean*	12.5 (1.9)	11.8 (2.1)	11.4 (2.3)	0.209	12.4 (2.0)	12.9 (1.4)	**0.001**
Gait speed, median (range)	0.83 (0.3)	0.61 (0.3)	0.8 (0.2)	**<0.001**	0.79 (0.2)	0.95 (0.3)	**<0.001**
Low gait speed, n (%)*	281 (29.1)	62 (65.3)	80 (36.0)	**<0.001**	47 (38.2)	92 (17.5)	**<0.001**
Handgrip strength, median (range)*	17.3 (8.0)	12.4 (5.8)	15.7 (7.1)	**<0.001**	16.0 (7.6)	19.3 (8.3)	**<0.001**
Low handgrip strength, n (%)*	779 (80.6)	94 (95.9)	188 (84.3)	**0.003**	102 (82.9)	395 (75.5)	0.081

**Table 2:** Association of gait speed and handgrip strength with falls in the participants with cognitive impairment. Abbreviations: CI; confidence interval, HGS; handgrip strength, GS; gait speed, OR; odds ratio. Model 1: unadjusted. Model 2: adjusted for age and sex. Model 3: adjusted for age, sex, cerebrovascular disease, depression, incontinence, polypharmacy, anticholinergic burden, and functional impairment. Values given in bold indicate statistically significant results (p < 0.05).

	Normal GS (n=175)		Low GS (n=142)			
	OR (95% CI)	p	OR (95% CI)	p		
Model 1	1 (Ref)	-	3.14 (1.90–5.12)	**<0.001**		
Model 2	1 (Ref)	-	2.60 (1.50–4.38)	**<0.001**		
Model 3	1 (Ref)	-	2.01 (1.10–3.77)	**0.019**		
	**Normal HGS (n=39)**		**Low HGS (n=282)**			
	OR (95% CI)	p	OR (95% CI)	p		
Model 1	1 (Ref)	-	4.01 (1.33–11.43)	**0.012**		
Model 2	1 (Ref)	-	3.23 (1.12–9.87)	**0.033**		
Model 3	1 (Ref)	-	3.57 (1.10–11.35)	**0.038**		
	**Normal GS and HGS (n=33)**		**Low GS or low HGS (n=143)**		**Low GS and low HGS (n=133)**	
	OR (95% CI)	p	OR (95% CI)	p	OR (95% CI)	p
Model 1	1 (Ref)	-	1.76 (0.55–5.49)	0.315	5.88 (1.90–17.46)	**0.001**
Model 2	1 (Ref)	-	1.52 (0.55–4.77)	0.440	4.33 (1.16–13.41)	**0.012**
Model 3	1 (Ref)	-	2.05 (0.62–7.18)	0.270	4.52 (1.14–15.78)	**0.024**

**Table 3:** Association of gait speed and handgrip strength with falls in the cognitively healthy participants. Abbreviations: CI; confidence interval, HGS; handgrip strength, GS; gait speed, OR; odds ratio. Model 1: unadjusted. Model 2: adjusted for age and sex. Model 3: adjusted for age, sex, marital status, education time, chronic obstructive lung disease, Parkinson’s disease, depression, osteoporosis, urinary incontinence, anticholinergic burden. Values given in bold indicate statistically significant results (p < 0.05).

	Normal GS (n=510)		Low GS (n=139)			
	OR (95% CI)	p	OR (95% CI)	p		
Model 1	1 (Ref)	-	3.20 (2.28–4.89)	**<0.001**		
Model 2	1 (Ref)	-	2.70 (2.10–4.51)	**<0.001**		
Model 3	1 (Ref)	-	2.13 (1.39–3.52)	**0.003**		
	**Normal HGS (n=149)**		**Low HGS (n=497)**			
	OR (95% CI)	p	OR (95% CI)	p		
Model 1	1 (Ref)	-	1.52 (0.93–2.57)	0.072		
Model 2	1 (Ref)	-	1.18 (0.70–2.01)	0.411		
Model 3	1 (Ref)	-	1.06 (0.63–1.98)	0.613		
	**Normal GS and HGS (n=132)**		**Low GS or low HGS (n=382)**		**Low GS and low HG (n=122)**	
	OR (95% CI)	p	OR (95% CI)	p	OR (95% CI)	p
Model 1	1 (Ref)	-	1.61 (0.94–2.98)	0.094	4.06 (2.03–7.67)	**<0.001**
Model 2	1 (Ref)	-	1.41 (0.79–2.48)	0.270	3.10 (1.58–6.05)	**0.022**
Model 3	1 (Ref)	-	1.11 (0.78–2.13)	0.540	1.67 (0.96–3.67)	0.120
